# Editorial: Molecular imaging for tracking drug delivery

**DOI:** 10.3389/fphar.2023.1195229

**Published:** 2023-05-18

**Authors:** Zheng Han, Tom Driedonks, Wei Tang, Zijian Zhou, Lacey McNally

**Affiliations:** ^1^ College of Medicine, University of Oklahoma Health Science Center, Oklahoma City, OK, United States; ^2^ Department of CDL Research, University Medical Center Utrecht, Utrecht, Netherlands; ^3^ Department of Pharmacy, National University of Singapore, Singapore, Singapore; ^4^ School of Biomedical Engineering, ShanghaiTech University, Shanghai, China

**Keywords:** molecular imaging, drug delivery, therapeutics, nanomedicine, treatment assessment

Molecular imaging provides novel opportunities to evaluate the efficacy of drug delivery, thereby enhancing the effectiveness of nanomedicine-based treatments (Kim et al., 2017). This Research Topic on *Molecular Imaging for Tracking Drug Delivery* showcases cutting-edge discoveries in nanoparticle development for drug delivery and their therapeutic effects. Simultaneously, it thoroughly discusses how different imaging methodologies in general can assist with drug delivery and treatment assessment.

We received multiple submissions for this Research Topic, of which five articles were finally included. These articles cover the latest developments in nanoparticle-based therapies for treatment of various diseases, ranging from arthritis, pulmonary fibrosis, spinal cord injury, post-myocardial infarction, and glioma. Besides describing the treatment progress, these articles also cover a broad range of imaging modalities that can be applied to monitor drug delivery *in vivo*, such as ultrasound and fluorescence imaging.

For brain tumor treatment, chemical exchange saturation transfer (CEST)-MRI has been a powerful tool to track drug delivery and its treatment effects on the molecular level (Huang et al., 2022). In this Research Topic, one review article thoroughly discussed the application of nanotechnologies for glioma treatment, which has the highest prevalence in malignant tumors of the central nervous system. Nanoparticles are reported to better control drug release, more easily pass the blood-brain barrier, and to be more degradable (Du et al.). This review article summarized the nanomaterials that can be used for a novel MRI technique and phototheranostic methods and highlights the impact of nanomedicine on glioma treatment based on the citation scores of publications.

All other four articles are original research papers. Compared to MRI, PET, and CT, ultrasound is fast and can be used for real-time imaging. It can also be used to monitor endovascular drug delivery (Wang et al., 2021). One of the articles in this Research Topic used ultrasound to guide intra-articular drug injection to treat antigen-induced arthritis (Li et al.). The researchers found that ultrasound can be an effective imaging tool to not only guide the nanoparticle injection, but also assess the treatment effects of the nanoparticles by providing ultrasonic scores of synovitis, synovial blood flow, and bone erosion. The authors show that both triptolide-loaded solid lipid nanoparticles and betamethasone achieved good results in treating arthritis for rabbits. Besides, ultrasound can also provide useful assessment of synovitis at early stages of arthritis.

The other three articles utilized fluorescence imaging, which is a fundamental tool for molecular imaging, biosensing, and treatment (Li et al., 2020). The researchers used fluorescence imaging to track extracellular vesicles (EVs), liposomes, and acetalated dextran (Ac-DEX) nanoparticles. One article in this Research Topic reports that EVs from cardiac progenitor cells can be tracked *in vivo* by near-infrared imaging (Roefs et al.). After intramyocardial injection, EVs can effectively migrate to the interstitial space of the myocardium and can interact with various types of cells in the heart. These results highlight cardiac progenitor cell-derived EVs as potential treatment for post-myocardial infarction. To further improve targeting, the researchers suggested decorating the EVs with targeting proteins to improve their interaction with cardiac endothelium, providing better therapeutics for the heart. Another featured study used a live imaging system and fluorescence imaging to track pirfenidone-containing, pH-sensitive liposomes (PSLs) during the treatment of idiopathic pulmonary fibrosis (Han et al.). In a rat model of bleomycin-induced pulmonary fibrosis, researchers found that pirfenidone PSLs inhibited the development of idiopathic pulmonary fibrosis, while the pirfenidone solution or phosphate-buffered saline were less effective. The fluorescence image clearly showed that pirfenidone PSLs accumulated in the lungs of rats with pulmonary fibrosis. The third study used immunofluorescence imaging to track paclitaxel-loaded Ac-DEX nanoparticles (Zhang et al.). It was shown the nanoparticles can be successfully delivered to the injury area of the spinal cord and can have sustained release of paclitaxel for up to 4 days. Moreover, in a rat model of spinal cord injury, the researchers found that these nanoparticles decreased the level of chondroitin sulfate proteoglycan, which can negatively impact tissue repair of injured nerves. This research opens new possibilities of using paclitaxel-loaded Ac-DEX to repair neurological injuries.

In conclusion, the collected articles in this Research Topic demonstrated the exciting development and applications of nanoparticles and nanomedicine for treatment of various types of complicated diseases. Molecular imaging methods, such as ultrasound, MRI, and fluorescence imaging, provide fundamental tools needed to track nanomedicines and assess their treatment effects. Empowered with the fast advancement of molecular imaging methods, we expect to see more exciting applications of novel nanomedicines towards health science.
